# Atypical Presentations of IPEX: Expect the Unexpected

**DOI:** 10.3389/fped.2021.643094

**Published:** 2021-02-05

**Authors:** Filippo Consonni, Sara Ciullini Mannurita, Eleonora Gambineri

**Affiliations:** ^1^Anna Meyer Children's Hospital, University of Florence, Florence, Italy; ^2^Division of Pediatric Oncology/Hematology, Meyer University Children's Hospital, Florence, Italy; ^3^Department of Neurosciences, Psychology, Drug Research, and Child Health (NEUROFARBA), University of Florence, Florence, Italy

**Keywords:** immune dysregulation, IPEX, regulatory T cells, *FOXP3*, primary immunodeficiencies

## Abstract

Immune dysregulation, polyendocrinopathy, and enteropathy, X-linked (IPEX) syndrome is a rare disorder that has become a model of monogenic autoimmunity. IPEX is caused by mutations in *FOXP3* gene, a master regulator of regulatory T cells (Treg). Cases reported in the last 20 years demonstrate that IPEX clinical spectrum encompasses more than the classical triad of early-onset intractable diarrhea, type 1 diabetes (T1D) and eczema. Atypical cases of IPEX include patients with late-onset of symptoms, single-organ involvement, mild disease phenotypes or rare clinical features (e.g., atrophic gastritis, interstitial lung disease, nephropathy etc.). Several atypical presentations have recently been reported, suggesting that IPEX incidence might be underestimated. Immunosuppression (IS) treatment strategies can control the disease, however at the moment allogeneic hematopoietic stem cell transplantation (HSCT) is the only available definitive cure, therefore it is important to achieve a prompt diagnosis. This review aims to describe unusual clinical phenotypes, beyond classical IPEX. Overall, our analysis contributes to increase awareness and finally improve diagnosis and treatment intervention in IPEX in order to ensure a good quality of life.

## Introduction

Immune dysregulation, polyendocrinopathy, enteropathy, X-linked (IPEX) syndrome is a rare congenital disorder caused by mutations in the Forkhead Box Protein 3 (*FOXP3*) gene, a master regulator of regulatory T cells (Treg). Its first clinical description dates back to 1982 (1), while its genetic characterization took place in 2000 (2, 3). Since then, research has shed light on its clinical spectrum and molecular features, and IPEX has become a model of monogenic autoimmunity and immune dysregulation (4). Moreover, recent advances proposed gene editing as a feasible therapeutic perspective for this syndrome (5), in addition to current treatments such as allogeneic hematopoietic stem cell transplantation (HSCT) and immunosuppression (IS) (6).

Although IPEX is a rare disease, more than 300 affected patients have been published so far (7), indicating an increasing awareness of the disorder (8). Cases reported in the last two decades demonstrate that IPEX clinical spectrum is much more heterogeneous, suggesting that its incidence might be underestimated (8). Apart from the classical triad (i.e., intractable diarrhea, type 1 diabetes mellitus—T1DM—and eczema), other autoimmune symptoms could also develop, such as thyroiditis, cytopenias, hepatitis, nephropathy or other (9). Interestedly, some reported patients only present with single organ involvement (10) or display unusual clinical features (11). Moreover, mild cases with late disease onset (12) and less severe disease course (13) have been described.

This review aims to focus on atypical clinical presentations of IPEX, giving examples of how the disease spectrum is wider than the usual classical manifestations. Mutations underlying atypical cases will be reported, with the aim of investigating possible genotype-phenotype correlations. Given the wide range of clinical manifestations, some “red flags” will be proposed. These could be helpful tools for clinicians in order to increase awareness of this disorder and to reduce diagnostic delay.

## Atypical IPEX: Clinical Phenotype

Classically, IPEX presents early in life and typical cases are characterized by the previously mentioned clinical triad often associated with failure to thrive (14). Diarrhea is due to autoimmune enteropathy, a hallmark of the disease, and it may precede or follow the onset of T1DM (8). Laboratory tests often show hypereosinophilia, elevated IgE levels and a wide variety of autoantibodies, not necessarily related to an ongoing autoimmune pathology. FOXP3 expression is variable in IPEX patients, depending on the type of mutations (4).

In the last two decades many reports have contributed to better define IPEX clinical spectrum and in particular to increase awareness of atypical presentations. Therefore, we performed an extensive literature review to summarize the main features of the atypical presentations based on cases reported. Accordingly, we propose to further classify atypical IPEX in cases characterized by late-onset (i.e., more than 1 year of age), mild disease course (i.e., long-term survival without IS or with first line IS regimens), no enteropathy and/or unusual clinical features (i.e., infrequent manifestations that go beyond the classical triad and/or involve different organs, therefore considered atypical for their unique presenting features).

### Late-Onset Disease

Recent studies show that the median age at disease onset is 2 months, even though its range is broad, going from prenatal manifestations to onset in the second decade of life (6). Rare cases of prenatal IPEX have been described, presenting toward the end of the second trimester of gestation with a lethal non-immune fetal hydrops (15, 16).

On the other hand, literature shows that IPEX can make its clinical debut later in life, the oldest reported patient being 12 years old at disease onset (12, 13). Enteropathy is the first manifestation in the majority of late-onset IPEX. However, a patient reported by Duclaux-Loras et al. displayed nephropathy as a first clinical sign and eventually died at 7 years (17), demonstrating that late-onset IPEX doesn't necessarily implicate a mild clinical phenotype. Classical IPEX features (i.e., diarrhea, dermatitis, and T1DM) have been largely described in patients with a delayed onset (6, 10, 11, 18–21). However, some patients with a mild clinical behavior together with a late clinical debut have been reported (12, 17). Interestingly, some late-onset IPEX patients had been initially treated as IBD (Inflammatory Bowel Disease) (10, 21, 22) and some received anti-TNF therapy with low clinical response. This confirms that IPEX should be suspected as a monogenic cause in front of a pediatric IBD phenotype (23), especially if treatment is not effective.

### Mild Disease

Recent reports revealed an increasing number of IPEX cases characterized by a less severe phenotype (e.g., mild eczema, well-tolerated diarrhea) (12, 17, 18, 24, 25). In these cases, symptoms are usually well-controlled by a single drug IS regimen (6). Moreover, in some patients no specific therapy is needed, except for hormone-replacement therapy if endocrine glands are involved (25). Mild IPEX cases are sporadic, if compared to patients with classical clinical features (17). However, such phenotypes have been increasingly described in recent years, possibly meaning that widespread use of genetic testing revealed several mild cases that had previously been underdiagnosed, since *FOXP3* sequencing was only reserved for severe clinical presentations.

As their severe counterpart, reported mild IPEX cases might also have an early clinical presentation. Enteropathy is usually the first presenting sign, even though Hwang et al. reported five *FOXP3*-mutated patients with early-onset T1DM, no gastrointestinal involvement and no need of IS (25). Laboratory tests show that immunoglobulin levels may vary from normal to reduced (12, 18), while IgE levels could be in range or increased (24). Autoantibodies are often detected, just like in severe forms. However, rare reports of mild cases with negative autoantibodies (17) suggest that this could possibly be considered a feature of mild IPEX. Anyhow, such finding needs to be confirmed in a larger cohort. Curiously, in Duclaux-Loras et al.'s cohort, Treg cells were measured in 10 patients and found normal in 3, two of whom were affected by mild disease (17). Such findings could reveal that mild IPEX patients have normal levels of Treg cells, potentially explaining the low intensity of the autoimmune phenomena in these cases. In the same study, authors speculated a relationship between mild phenotypes and mutations within the first splice donor site (17). Such genotype-phenotype correlation will be furtherly discussed.

### No-Enteropathy

Interestingly, some cases of IPEX without enteropathy are reported (24–29). In these patients, first disease manifestation is usually early-onset T1DM (25). Even in the presence of diabetes, however, diagnostic suspicion could be jeopardized by the absence of enteropathy since intractable diarrhea is universally known as a hallmark of IPEX syndrome (27). For the same reason, IPEX cases without enteropathy could be underdiagnosed.

### Unusual Clinical Features

**Figure 1:**
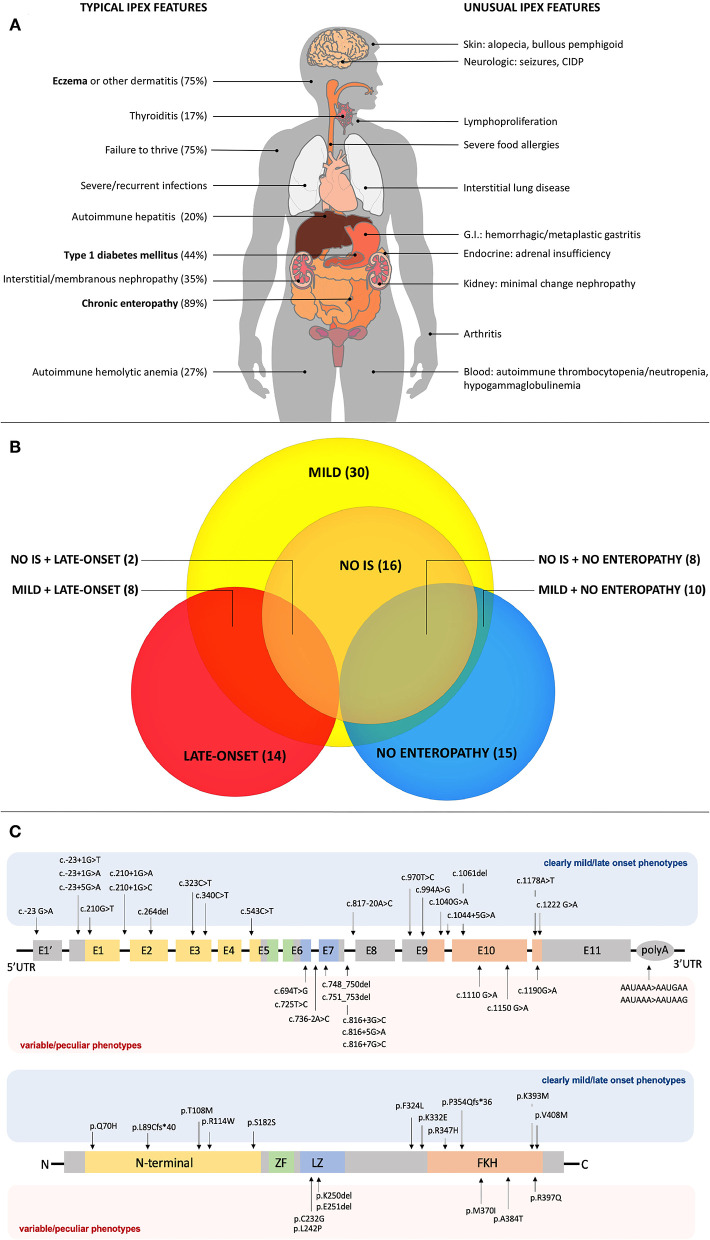
**(A)** Typical and unusual clinical features in IPEX. Percentages for typical features are based on the most recently published IPEX cohort (6). Classical triad features are in bold. CIDP, Chronic Inflammatory Demyelinating Polyneuropathy; GI, Gastrointestinal. **(B)** Relationships among atypical IPEX subgroups revealed by our analysis. Subgroup (number of patients); IS, Immunosuppression. **(C)** *FOXP3* gene and protein structure showing mutations associated with mild/late-onset and variable/peculiar IPEX phenotypes.

Reported cases of IPEX show an increasing number of additional clinical manifestations beyond the classical triad (4) (Figure 1A). Apart from pancreatic islets in T1DM, other endocrine organs can be involved. While the thyroid gland is the second most frequently affected one (9), rare cases of adrenal insufficiency are also reported (26). Together with eczema, infrequent cutaneous manifestations include psoriasiform and ichthyosiform dermatitis (30), alopecia (31) and bullous pemphigoid (32). Similarly, enteropathy can have different histopathologic phenotypes (i.e., GvHD-like, depletion of goblet cells and Coeliac Disease-like) (19). Notably, Coeliac Disease (CD) antibodies can also be detected. For this reason, patients with early-onset enteropathy and positive CD serology who do not respond to gluten-free diet should raise suspicion of IPEX, even in the presence of CD-like histologic findings (13). Another unusual gastrointestinal finding is gastritis, which is usually atrophic (33) and might display hemorrhagic features (34) or metaplastic epithelial changes (11).

Several reported cases of IPEX developed kidney involvement, which can infrequently be the first clinical manifestation (29). Membranous glomerulonephritis and interstitial nephritis are the most commonly reported (4, 29, 35, 36), but also Minimal-Change Nephropathy (MCN) has been described (37, 38). Anyhow, renal involvement is not only caused by underlying autoimmune processes, since IS drugs used in IPEX can induce nephrotoxicity (4). Similarly, lung involvement has been described, even though it is sometimes difficult to ascertain whether it is due to infections or to autoimmunity (4, 39). Anyhow, an autoimmune pneumopathy can be inferred if clinical signs improve with IS treatment. Such cases have been occasionally reported and are associated with fatal outcomes (39).

AutoImmune Hemolytic Anemia (AIHA) is frequent [27% of cases in a recent cohort (6)], while thrombocytopenia and neutropenia are quite unusual (4). Cytopenias can rarely coexist (29), therefore IPEX should be considered as a potential underlying cause of Evans syndrome (40) together with other Tregopathies such as CTLA-4 haploinsufficiency, LRBA, STAT3-GOF etc. (41–43). Similarly, autoimmune hepatitis is not rare, being reported in ~20% of cases (6), and it can present both with both positive and negative autoantibodies (29, 44). Signs of lymphoproliferation are occasionally described, such as splenomegaly, lymphadenopathy and lymphocytic infiltrates in multiple organs (14, 45). Finally, infrequent—yet reported—clinical findings are arthritis [whose severity and extension are quite variable (13, 45)] and severe food allergies, which can further complicate gastrointestinal symptoms (46).

## Atypical IPEX: Genotype-Phenotype Correlations

Overall, our analysis of atypical forms revealed 30 mild, 14 late-onset and 15 cases with no enteropathy (Figure 1B). Moreover, several unusual clinical manifestations have been identified (Tables 1–**3**). Even though IPEX so far revealed an inconsistent genotype-phenotype correlation (9), we have clustered in this section reported cases that meet the criteria above, indicating—when available—information about FOXP3 protein expression and T_reg_ percentage.

**Table 1:** Mutations clearly associated with a mild/late-onset IPEX phenotype.

**Pt**	**Mutation**	**Location**	**Mut Type**	**Mutation effect**	**Entero-pathy**	**Mild**	**Late onset**	**Other clinical features**	**Notable Lab features**	**Definitive Treatment**	**References**
1	c.−23G>A	1st donor splice	SP	Incorrect initiation codon. Low FOXP3	yes	no	no	**Onset with membranous and interstitial nephropathy**, T1DM, dermatitis		HSCT	(29)
2	c.−23+1G>T	1st donor splice	SP	Incorrect initiation codon. Low FOXP3 and Treg	yes	**yes**	no	Nephrotic syndrome, T1DM		CsA, Ster	(47)
3	c.−23+1G>A	1st donor splice	SP		yes	**yes**	no	**Mild enteropathy**, dermatitis, severe food allergy	No auto-Abs	**No IS**	(17)
4	c.−23+5G>A	1st donor splice	SP	Normal Treg	yes	**yes**	no	**Mild enteropathy**, T1DM, dermatitis, **arthritis**		**No IS**	(17)
5	c.−23+5G>A	1st donor splice	SP	Low FOXP3, normal Treg	yes	**yes**	**yes**	T1DM, dermatitis, coeliac disease		**No IS**	(17)
6	c.210+1G>A	N term	SP		**no**	no	no	T1DM, membranous nephropathy, **PRCA, PRES**, meningitis		Ster	(27)
7	c.210+1G>A	N term	SP		**no**	no	no	Dermatitis, nephropathy		na	(44)
8	c.210+1G>C	N term	SP	Low FOXP3	yes	**yes**	**yes**	T1DM, nephropathy, lymphadenopathy, FTT		Rapa	(6)
9	c.210G>T, p.Q70H	N term, PRR	MS		yes	**yes**	no	Dermatitis, Infections, ITP, FTT	**HGG**	IVIG, Rapa	(21)
10	c.264del, p.L89Cfs*40	N term, PRR	FS	Premature stop codon	yes	no	**yes**	**Onset with interstitial nephropathy, late-onset enteropathy**, T1DM, exitus		HSCT	(17)
11	c.323C>T, p.T108M	N term, PRR	MS	Normal CD4+CD25+, loss of Treg suppression	yes	**yes**	**yes**	**Arthritis, pleuritis, pericarditis**		AZA, Ster	(13)
12	c.340C>T, p.R114W	N term, PRR	MS		**no**	**yes**	no	T1DM, dermatitis		**No IS**	(25)
13	c.817-20A>C	Upstream ex 8	SP		yes	no	**yes**	**Misdiagnosed as IBD**, AKI due to CsA, dermatitis		Rapa, MTX	(22)
14	c.970T>C, p.F324L c.543C>T, p.S182S	Upstream FKH	MS	Normal FOXP3, normal Treg suppression	yes	**yes**	no	Sibling pt 15. Dermatitis, nephropathy		Rapa,Ster	(48)
15	c.970T>C, p.F324L c.543C>T, p.S182S	Upstream FKH	MS	Normal FOXP3, normal Treg suppression	no	**yes**		Sibling pt 16. **Healthy**		**No IS**	(48)
16	c.994A>G, p.K332E	FKH	MS		yes	**yes**	**yes**	T1DM, dermatitis, **lung involvement**, FTT		Rapa	(24)
17	c.1040G>A, p.R347H	FKH	MS	Normal FOXP3	no	**yes**	no	T1DM		**No IS**	(28)
18	c.1040G>A, p.R347H	FKH	MS		no	**yes**	no	T1DM, dermatitis, FTT		**No IS**	(25)
19	c.1040G>A, p.R347H	FKH	MS	Low FOXP3	yes	**yes**	no	**Mild enteropathy**, T1DM, dermatitis, **hepatitis, EPI**, ITP		**No IS**	(33)
20	c.1040G>A, p.R347H	FKH	MS	Normal Treg suppression	yes	no	no	T1DM, dermatitis**, atrophic gastritis, EPI**, recurrent otitis		CsA, Ster	(33)
21	c.1040G>A, p.R347H	FKH	MS	Normal Treg	yes	**yes**	**yes**	Dermatitis		Rapa	(12)
22	c.1040G>A, p.R347H	FKH	MS		**no**	no	no	T1DM, **hemorrhagic gastritis**, bronchiectasis, FTT	HGG	CsA, Ster	(34)
23	c.1040G>A, p.R347H	FKH	MS		**yes**	yes	no	T1DM, dermatitis, hepatitis: all but T1DM regressed at 26 y/o		No IS	(49)
24	c.1044+5G>A	FKH	SP	Skipping ex 10, normal FOXP3	**no**	**yes**	no	Sibling pt 24. T1DM, dermatitis		**No IS**	(25)
25	c.1044+5G>A	FKH	SP	Skipping ex 10, normal FOXP3	**no**	**yes**	no	Sibling pt 23. T1DM, dermatitis, FTT, severe infections.		**No IS**	(25)
26	c.1044+5G>A	FKH	SP		**no**	**yes**	no	T1DM, ADHD		**No IS**	(25)
27	c.1061del, p.P354Qfs*36	FKH	FS	Premature stop codon	yes	**yes**	**yes**	**Misdiagnosed as IBD**, dermatitis		Rapa, Ster	(21)
28	c.1178A>T, p.K393M	FKH	MS		yes	**yes**	no	**Mild enteropathy**, T1DM, dermatitis, infections		**No IS**	(25)
29	c.1222 G>A, p.V408M	FKH	MS	Reduced flexibility of DBD	**no**	**yes**	no	T1DM, **MCN**, Transient Ischemic Attack		Ster	(50)
30	c.1222 G>A, p.V408M	FKH	MS	Reduced flexibility of DBD	yes	**yes**	no	Sibling pt 30. **Late-onset mild enteropathy**. T1DM at 3 weeks, Hypothyroidism, infections, candidiasis, mild ID		na	(50)
31	c.1222 G>A, p.V408M	FKH	MS	Reduced flexibility of DBD	yes	**yes**	no	Sibling pt 29. **Late-onset mild enteropathy**. T1DM at 3 months, Hypothyroidism, infections, candidiasis, mild ID		na	(50)

*Pt, Patient; Mut, Mutation; SP, Splicing; MS, Missense; FS, Frameshift; DBD, DNA binding domain; FKH, Forkhead domain; PRR, Proline-rich region; ex, exon; T1DM, Type 1 diabetes mellitus; PRCA, Pure Red Cell Aplasia; PRES, Posterior Reversible Encephalopathy Syndrome; FTT, Failure to thrive; ITP, Idiopathic Thrombocytopenic Purpura; AKI, Acute Kidney Injury; EPI, Exocrine Pancreatic Insufficiency; ADHD, Attention Deficit and Hyperactivity Disorder; MCN, Minimal Change Nephropathy; ID, Intellectual Disability; HGG, Hypogammaglobulinemia; HSCT, Hematopoietic Stem Cell Transplantation; CsA, Cyclosporine A; Ster, Steroid; IS, Immunosuppression; Rapa, Rapamycin/Sirolimus; IVIG, Intravenous Immunoglobulins; AZA, azathioprine; MTX, Methotrexate; na, not available. Clinical features considered most noteworthy are in bold*.

As shown in Table 1, some *FOXP3* mutations seem to be clearly associated with atypical phenotypes. Conversely, other mutations may bear from mild to severe presentations with a still debated genotype-phenotype association. Therefore, we have defined these mutations as associated with a variable disease course (Table 2). Finally, some peculiar features have shown to be recurrent in patients carrying certain mutations: these cases have been depicted in Table 3. We reported *FOXP3* gene and corresponding protein mutations associated with the described phenotypes in Figure 1C.

**Table 2:** Mutations associated with a variable IPEX phenotype.

**Mutation**	**Location**	**Mut Type**	**Mutation effect**	**Enteropathy**	**Mild**	**Late onset**	**Relevant clinical features**	**Notable lab features**	**Definitive treatment**	**References**	***N* of Pts reported**
c.694T>G, p.C232G	LZ	MS	Normal/low FOXP3, normal/poor Treg suppression	yes	**var**	**var**	**Recurrent sinopulmonary infections, cutaneous candidiasis**, arthritis, T1DM, dermatitis, AIHA, FTT	**HGG**, B cell class switching defect	Variable: no IS, Rapa + ster, HSCT	(18, 51)	7
c.725 T>C, p.L242P	LZ	MS	Normal FOXP3	**var**	**var**	no	T1DM, dermatitis, AIHA, nephropathy, sepsis		na	(9, 24)	2
c.736-2A>C	Intron 7	SP	Skipping ex 7, low FOXP3	**var**	**var**	no	**Membranous nephropathy, MCN**, T1DM, dermatitis, infections, hypothyroidism, AIHA, ITP, FTT		CsA, Tacrolimus, Rituximab, ster	(37, 52)	3
c.748_750del, p.K250del	LZ	IFD	Low FOXP3	yes	**var**	**var**	**Atrophic/metaplastic gastritis, MCN, hepatitis, arthritis**, AIHA, ITP, food allergies, T1DM, dermatitis, FTT		Variable: 6-MP, CsA + ster, HSCT	(11, 38, 45)	4
c.1150 G>A, p.A384T	FKH	MS	Low FOXP3, low Treg suppression	**var**	**var**	no	**Mild enteropathy**, dermatitis**, alopecia, adrenal dysfunction**, FTT		Variable: No IS, Tacrolimus + ster	(24, 26)	3
c.1190G>A, p. R397Q	FKH	MS	Low CD4+CD25+	yes	no	**yes**	**Misdiagnosed as IBD**, T1DM, dermatitis, infections, candidiasis, AIHA, food allergies, FTT	Low CD4, decreased proportions of CD19 cells	HSCT	(10, 31, 44)	3
AAUAAA>AAUGAA	PolA	PolA	Unstable mRNA	**var**	no	no	T1DM, dermatitis, AIHA, **adrenal insufficiency, nephropathy**, food allergy, FTT, infections		Variable: HSCT, Tacrolimus + ster	(3, 24)	6
AAUAAA>AAUAAG	PolA	PolA	Unstable mRNA and absence of Treg	yes	no	no	T1DM, dermatitis, **lung involvment, lymphadenopathy**, AIHA, ITP, FTT		Variable: ster, IVIG, HSCT	(24, 53)	3

*Mut, Mutation; LZ, leucine Zipper domain; FKH, Forkhead domain; PolA, Polyadenilation signal sequence; MS, Missense; SP, Splicing; IFD, In-frame deletion; ex, exon; var, variable; T1DM, Type 1 diabetes mellitus; AIHA, Autoimmune hemolytic anemia; FTT, Failure to thrive; MCN, Minimal Change Nephropathy; ITP, Immune thrombocytopenic purpura; HGG, Hypogammaglobulinemia; cTFH, circulating T Follicular Helper cells; IS, Immunosuppression; Rapa, Rapamycin/Sirolimus; ster, Steroid; HSCT, Hematopoietic Stem Cell Transplantation; CsA, Cyclosporin A; 6-MP, Mercaptopurine; IVIG, Intravenous immunoglobulins; na, not available. Clinical features considered most noteworthy are in bold*.

**Table 3:** Mutations associated with peculiar clinical features of IPEX.

**Mutation**	**Location**	**Mut Type**	**Mutation effect**	**Enteropathy**	**Mild**	**Late onset**	**Relevant clinical features**	**Notable Lab features**	**Definitive Treatment**	**References**	***N* of Pts reported**
c.210+1G>A	N term	SP		**var**	no	no	T1DM, dermatitis, **nephropathy**, AIH, thyroiditis		HSCT	(44)	2
c.210+1G>C	N term	SP	Low FOXP3	yes	no	**yes**	T1DM, **nephropathy**, lymphadenopathy, FTT		Rapa	(6)	1
c.694T>G, p.C232G	LZ	MS	Normal/low FOXP3, normal/poor Treg suppression	yes	**var**	**var**	**Recurrent sinopulmonary infections, cutaneous candidiasis**, arthritis, T1DM, dermatitis, AIHA, FTT	**HGG**, B cell class switching defect, Th1/Th17 skewing of cTFH	Variable: no IS, Rapa + ster, HSCT	(18, 51)	7
c.736-2A>C	Intron 7	SP	Skipping ex 7, low FOXP3	**var**	**var**	no	**Membranous nephropathy, MCN**, T1DM, dermatitis, infections, hypothyroidism, AIHA, ITP, FTT		CsA, Tacrolimus, Rituximab, ster	(37, 52)	3
c.751_753del, p.E251del	LZ	IFD	Absent FOXP3	yes	no	**var**	**Late-onset enteropathy with depletion of goblet cells**, T1DM, dermatitis, nephropathy, AIHA, ITP, **neutropenia**, hypothyroidism, **CIDP**, AIH, FTT	Anti-goblet cells Abs	HSCT	(19, 29, 54)	4
c.816+ 3G>C	downstream ex 7	SP	Low FOXP3	yes	no	no	Dermatitis, **nephropathy**, thrombosis, fractures, infections, FTT		HSCT	(24)	1
c.816+5G>A	downstream ex 7	SP	Normal FOXP3, normal Treg suppression	yes	no	no	Thyroiditis, **adrenal insufficiency, seizures**, infections, **nephropathy, HSM, lymphadenopathy**, **lung involvement**, AIH, arthritis, **neutropenia**, T1DM, dermatitis, FTT	**HGG**, high IgE	Variable: CsA, Rapa, IVIG, ster, AZA, HSCT	(24, 39)	6
c.816+7G>C	downstream ex 7	SP	Skipping ex 7, Normal FOXP3	yes	no	no	T1DM, dermatitis, **membranous nephropathy, ILD, seizures**, **GH deficiency**, hypothyroidism, infections, FTT	**HGG**, high IgE	HSCT	(24, 29, 55)	4
c.1110 G>A, p.M370I	FKH	MS	Low FOXP3 Treg	yes	no	no	Dermatitis, **HSM, lymphadenopathy**, nephropathy, lung involvement, AIHA, ITP, **neutropenia**, AIH, FTT		na	(24, 56)	2

*Mut, Mutation; LZ, leucine Zipper domain; FKH, Forkhead domain; SP, Splicing; MS, Missense; IFD, In-frame deletion; ex, exon; var, variable; T1DM, Type 1 diabetes mellitus; FTT, Failure to thrive; AIHA, Autoimmune hemolytic anemia; ITP, Immune thrombocytopenic purpura; CIDP, Chronic inflammatory demyelinating polyneuropathy; AIH, autoimmune hepatitis; HSM, hepatosplenomegaly; ILD, Interstitial lung disease; GH, Growth hormone; HGG, Hypogammaglobulinemia; IS, Immunosuppression; Rapa, Rapamycin/Sirolimus; ster, Steroid; HSCT, Hematopoietic Stem Cell Transplantation; CsA, Cyclosporin A; 6-MP, Mercaptopurine; IVIG, Intravenous immunoglobulins; AZA, Azathioprine; na, not available. Clinical features considered most noteworthy are in bold*.

### Mutations Clearly Associated With Atypical Phenotypes

#### Mutations in Non-coding Regions

Mutations associated with mild IPEX phenotypes tend to occur outside *FOXP3* coding regions (Table 1 and Figure 1C). Genetic defects located within an intron/exon splice junction or in the first polyadenylation signal might hamper gene expression and protein production (17). Besides, the first splice donor site is highly methylated due to the presence of multiple conserved non-coding enhancer sequences responsible for epigenetic regulation (57). Mutations located in this site might affect the overall methylation status leading to a decreased FOXP3 expression, thus resulting in an atypical phenotype (17).

In detail, 5 patients with c.−23+1G>A or c. −23+5G>A mutations located in the first spice site are reported: 4 out of 5 suffered from a mild disease, and 3 never needed IS therapy (17, 29, 47, 58). Interestingly, laboratory studies in these patients show a variable Treg expression, two of them had normal Treg counts but a low FOXP3 expression.

Similarly, c.1044+5G>A mutation was identified in 3 patients who did not develop enteropathy, nor needed IS therapy. This genetic defect is located between exons 9 and 10, both coding for the DNA-binding FKH domain. Molecular studies showed that this mutation affects RNA splicing, inducing the formation of both a wild-type and a truncated transcript lacking exon 10. It is therefore not surprising that FOXP3 protein expression in CD4^+^ T cells is normal in these cases. Two other splicing mutations (c.210+1G>C and c.817-20A>C) were described in patients with late-onset enteropathy, showing that a mild phenotype is also associated with a delayed disease onset.

#### Mutations in Coding Regions

Nevertheless, also mutations in coding regions appear to be correlated to atypical phenotypes. Both missense and frameshift types of mutations have been clearly associated with atypical IPEX.

Above all, mild or unusual cases of IPEX caused by the missense variant c.1040G>A (p.R347H) have been repeatedly described (12, 24, 25, 28, 33, 34). Functional studies showed that mutant cells have a preserved ability to suppress cytokine production on CD4+ T cells (33), while a reduced CD25 expression has been reported only in some patients with this genotype (12, 33). Even though this mutation can also cause severe IPEX, affected patients displayed anyhow unusual clinical features [e.g., atrophic or hemorrhagic gastritis (33, 34)] or—interestingly—severe manifestations regressed at the suspension of IS in adulthood (49).

Additional reports of missense mutations causing mild phenotypes were published for c.210G>T (p.Q70H), c.340C>T (p.R114W), c.1178A>T (p.K393M), c.1222G>A (p.V408M) and c.970T>C (p.F324L) (6, 25, 27, 44, 48, 50, 59). Interestingly, the latter was described in a healthy male whose brother was affected by IPEX (24). Both patients also presented the synonymous variant c.543C>T (p.S181S), already identified in other healthy subjects (24). Laboratory studies showed normal CD25 and FOXP3 expression, and a preserved Treg suppressive ability. This defect involves a coding region, therefore a possible explanation for this behavior is that both phenylalanine and leucine are hydrophobic, and their substitution does not affect the protein's tertiary structure. Alternatively, other genetic or epigenetic features could contribute to stabilize FOXP3 structure and guarantee Treg function.

Finally, two frameshift mutations have been reported in two late-onset cases of IPEX: c.1061delC (p.P354Qfs^*^36) and c.264delC (p.L89Cfs^*^40) (17, 21). Frameshift mutations, which totally alter the protein's primary structure, can still give rise to late-onset clinical findings. c.264delC is located in exon 2, which can physiologically be alternatively spliced, generating exon 2^minus^ transcripts (24). Molecular studies are not available for these cases, but further research could show if such exon 2-mutated Tregs could maintain their correct functionality.

### Mutations Associated With a Variable Phenotype

While on one hand some genotypes have been repeatedly associated with mild clinical manifestations, another set of mutations is not clearly related to a precise phenotype and a possible genotype-phenotype correlation needs to be further elucidated. These mutations are therefore associated with variable disease phenotypes (Figure 1C and Table 2).

#### Mutations in the Leucine-Zipper Domain

Four mutations associated with a variable disease phenotype involve the leucine zipper domain, required for FOXP3 homodimerization and transcriptional activity; these are: c.694T>G (p.C232G), c.725T>C (p.L242P), c.736-2A>C and c.748_750del (p. K250del). Therefore, impaired formation of FOXP3 homo-/heterodimers in Treg cells both diminishes FOXP3 functions and destabilizes its expression. This has been particularly elucidated for the intronic mutation c.736-2A>C (52), responsible for exon 7 skipping. All three patients with this mutation displayed membranous nephropathy or MCN. Indeed, *FOXP3* transcripts lacking exon 7 have been associated with a Th17 differentiation, as described in patients with multifactorial autoimmune diseases such as rheumatoid arthritis and Crohn's disease (60). Further research is needed to ascertain if autoimmune manifestation (e.g., nephropathy) in IPEX patients carrying this mutation are driven by Th17 cells.

Several cases of c.694T>G (p.C232G) are reported in literature. Among these is a family cluster displaying a Common Variable Immunodeficiency-like (CVID-like) phenotype, which has rarely been described in IPEX (18). On the other hand, the same mutation has been reported in other 3 patients, who presented a more severe phenotype, though still displaying susceptibility to infections (51). Laboratory tests revealed low FOXP3^+^ Tregs in the first family, while FOXP3 expression was normal in the patients reported in the second manuscript.

#### Mutations in the DNA-Binding Domain

c.1150G>A (p.A384T) and c.1190G>A (p.R397Q) are the only two mutations involving the DNA-binding domain and determining a variable disease phenotype (26). The former has been shown to impair suppressive Treg function, while preserving FOXP3 ability to repress the production of inflammatory cytokines. This is possibly due to disruption of *FOXP3* binding to histone acetyltransferase (61). On the other hand, a severe IPEX phenotype has once been reported for p.A384T (24), therefore its association with a mild disease phenotype is still debated and needs to be confirmed by further reports.

#### Mutations in PolyA Sequence

Several cases of mutations in the mRNA polyadenylation (PolyA) signal sequence have been described (3, 24, 53), though still underestimated, since such region is frequently neglected in usual sequencing approaches. PolyA sequence protects transcripts from degradation, and patients with these defects have low-level expression of FOXP3 (3). On the other hand, a high variability characterizes these cases, since phenotypes range from an incomplete triad to severe or unusual manifestations (e.g., adrenal insufficiency and pneumopathy). Such clinical diversity could be related to a variable amount of transcript degradation and mRNA stability in the cytoplasm (24).

### Mutations Associated With Peculiar Clinical Features

Some *FOXP3* mutations seem to be associated with precise organ involvement (i.e., nephropathy), specific laboratory alterations (i.e., hypogammaglobulinemia) or other peculiar clinical findings (Figure 1C and Table 3).

#### Nephropathy-Correlated Mutations

c.210+1G>A and c.736-2A>G splicing mutations are repeatedly associated with nephropathy. As previously described, kidney involvement is not rare in IPEX and could be due both to autoimmunity and to IS drugs side effects (29). In detail, three patients with c.736-2A>G displayed either membranous glomerulopathy or MCN, both of which are caused by an underlying autoimmune process. Kidney involvement should be suspected when facing patients with this splicing mutation, even though precise genotype-nephropathy correlations still need to be elucidated.

#### Hypogammaglobulinemia-Correlated Mutations

Hypogammaglobulinemia has been described in several IPEX cases carrying the c.694T>G (p.C232G) mutation (18, 51). Cysteine 232 is located immediately upstream of the leucine zipper domain and could therefore strongly impact on FOXP3 protein dimerization ability and its interaction with other transcription factors. Even though functional studies showed that Treg suppressive ability may be either suppressed or preserved (51), Shamriz et al. demonstrated important immunological consequences of this mutation (18). These include B cell class switching defect and Th1/Th17 skewing of cTFH (circulating T Follicular Helper) cells. Such findings may potentially explain the clinical findings displayed by patients with this defect (i.e., recurrent infections, candidiasis).

#### Other Peculiar Clinical Findings

Intriguingly, unusual clinical features have been repeatedly described in patients with c.1110G>A (p.M370I) lymphoproliferation) (24, 56) and c.751_753del (p. E251del, neutropenia, chronic inflammatory demyelinating polyneuropathy and other) (54). Finally, the exon 7-skipping mutations c.816+3/5/7G>C have been described in several patients. None of them had a mild or late-onset phenotype, while several unusual manifestations were reported (i.e., hypogammaglobulinemia, seizures, autoimmune pneumopathy) (24, 39, 55). As previously described, skipping exon 7 may be associated with an increased Th17 differentiation (60). Further evidence is needed to elucidate if such immunologic features underlie these unusual clinical presentations.

## Conclusion

IPEX is a multisystem autoimmune disease, characterized by a universe of heterogeneous clinical manifestations that trespass the classical phenotype. Widespread use of genetic testing raised our awareness of atypical disease presentations. Such knowledge allowed us to unmask atypical IPEX cases, revealing that the real incidence of the disease might be underestimated. Herein we highlight the importance of picking up unusual signs of IPEX in order to facilitate physicians to suspect the disease even when canonical clinical findings are not fully respected (Box 1).

Box 1 **Red flags for early recognition of atypical IPEX.**

- Gastrointestinal
  ◦ Chronic/intermittent diarrhea resistant to formula switching
  ◦ Early-onset IBD or treatment-resistant IBD
  ◦ Coeliac disease not responding to gluten-free diet
- Endocrine
  ◦ Late onset type 1 diabetes mellitus
- Dermatitis associated with other autoimmune features
- Multiorgan autoimmunity with or without enteritis
- Do not exclude even if onset is in late childhood

Genotype-phenotype correlation is still unclear, but our analysis shed light on some mutations more frequently associated with a mild phenotype. Intronic mutations located in the first donor splice site seem to have a significant role; however, other genetic defects located in non-coding and coding regions of *FOXP3* can result in atypical disease (Figure 1C). For instance, mutations in the DNA-binding site, a FOXP3 functional domain, classically associated with poorer survival (17), have also been related with moderate clinical manifestations. Nevertheless, the complexity of interactions of *FOXP3* with other genes and its epigenetic regulation can also be responsible for phenotype variability, rather than *FOXP3* variants themselves. Thus, further studies are needed to further elucidate other contributing mechanisms. Similarly, future focus on other actors such as Type 1 regulatory cells (Tr1) and on the plasticity of the immune system may reveal other intriguing aspects of IPEX immunopathogenesis (62).

Overall, our analysis contributes to increase awareness and to improve diagnosis and treatment intervention in IPEX. Notably, 16 reported patients are clinically stable without any IS regimen, showing that IPEX therapeutic scenarios may range from these cases to invasive options such as allogeneic HSCT. Meanwhile, novel gene therapy-based approaches are still under study but may definitively change the natural history of this disorder (5).

## Author Contributions

SC and FC reviewed literature and performed analysis of data. EG supervised the work and wrote the original draft of the article. All authors contributed to the article and approved the submitted version.

## Conflict of Interest

The authors declare that the research was conducted in the absence of any commercial or financial relationships that could be construed as a potential conflict of interest.
